# How generative AI enhances self-regulated learning in EFL learners: a chain mediation model of “intention to use” and “learning engagement”

**DOI:** 10.3389/fpsyg.2026.1808183

**Published:** 2026-04-01

**Authors:** Jin Xu

**Affiliations:** General Education College, Jinhua University of Vocational Technology, Jinhua, Zhejiang, China

**Keywords:** EFL (English as a Foreign Language), generative AI, learning engagement, self-regulated learning, technology acceptance model

## Abstract

Generative Artificial Intelligence is transforming English as a Foreign Language writing instruction. While much research has focused on technology acceptance, limited attention has been paid to how AI influences learners’ psychological and behavioral processes, thereby affecting self-regulated learning. This study constructs and tests a theoretical model that examines the chain mediation of “intention to use → learning engagement” to explore how generative AI impacts self-regulated learning in EFL learners. Involving 386 Chinese EFL learners from universities, the study uses a questionnaire survey and structural equation modeling for data analysis. The findings show that the perceived ease of use, usefulness, and interactivity of generative AI significantly predict both intention to use and self-regulated learning. Intention to use is related to learning engagement, which in turn fosters self-regulated learning. Moreover, generative AI enhances self-regulated learning through intention to use and learning engagement, with these factors serving as key mediators. This study illuminates the pathway from “technology perception” to “behavioral intention,” “immersive engagement,” and “regulatory process,” providing novel insights into AI-assisted second language writing learning and offering empirical evidence for designing effective human-computer collaborative EFL writing practices.

## 1 Introduction

Recent advances in generative artificial intelligence (GAI), exemplified by tools such as ChatGPT, are significantly reshaping educational landscapes, offering both unprecedented depth and breadth. In the domain of computer-assisted language learning (CALL), particularly in English as a Foreign Language (EFL) writing instruction, these innovations have introduced a new paradigm of AI-assisted writing (Kohnke et al., 2023; Wulyani et al., 2024). By harnessing advanced natural language processing and content generation capabilities, generative AI provides substantial support to EFL writers, facilitating the entire writing process—from idea generation and text production to feedback provision and refining linguistic accuracy (Liu et al., 2024). Consequently, generative AI shows potential for enhancing EFL writing instruction, offering benefits such as higher writing fluency and greater learner engagement (Wei et al., 2023; Chan et al., 2024; Bueie et al., 2025).

In response to this technological shift, early empirical research has predominantly focused on learners’ acceptance of technology. Drawing on frameworks such as the Technology Acceptance Model (TAM), a growing body of studies has explored how learners’ individual characteristics and their perceptions of generative AI influences their intention to adopt the technology for language learning (Chen et al., 2024; Firdaus et al., 2024). However, technology adoption represents only the initial stage of technology-enhanced learning. Relatively little attention has been given to how generative AI is integrated into actual writing practices and how it reshapes learners’ psychological and behavioral processes, ultimately influencing higher-order learning outcomes, such as self-regulated learning (SRL).

Existing studies have largely focused on isolated relationships, such as the direct effects of AI use on learning engagement (Wang and Xue, 2024) or self-regulated learning (Liu et al., 2024). While some research has examined the link between learning engagement and SRL (Yang and Du, 2025), these constructs are rarely integrated within a coherent theoretical model that empirically tests their underlying mechanisms. Specifically, the processes through which learners’ perceptions of generative AI translates into adoption intention, fosters deeper learning engagement, and ultimately facilitates the development of self-regulated learning remain largely unexplored. This presents a critical challenge to understanding how generative AI can effectively support language learning beyond initial adoption.

This gap is of particular theoretical significance when viewed through the lens of self-regulated learning (SRL) theory. According to the social cognitive model of SRL, environmental factors (e.g., technological tools) influence SRL by interacting with learners’ behavioral processes (e.g., engagement) and personal factors (e.g., beliefs and motivation) (Zimmerman, 2002). In parallel, Fredricks et al. (2004) conceptualize learning engagement as a critical mediator linking instructional interventions to learning outcomes. From this perspective, integrating TAM with SRL theory and explicitly modeling the “intention to use → learning engagement” chain mediation process is essential for uncovering the psychological mechanisms through which generative AI supports language learning.

To address both the theoretical and empirical gaps, this study goes beyond a simple “technology–outcome” relationship and explores the central question: How do generative AI used in EFL writing impacts learners’ self-regulated learning through their intention to use and engagement? Specifically, this research develops and empirically tests a chain mediation model that outlines the complete pathway, from technological perceptions (such as perceived ease of use, perceived usefulness, and perceived interactivity) to behavioral intention (intention to use), through immersive involvement (learning engagement), and ultimately to regulatory processes (self-regulated learning).

By integrating the TAM and SRL frameworks, this study provides novel insights into how generative AI influences self-regulated learning through interconnected psychological processes. The findings offer a new perspective on learning in human–AI collaborative environments and provide empirical guidance for EFL teachers and instructional designers on how to optimize AI tool features and design pedagogical activities that guide learners from technology acceptance, through deep engagement, to the development of self-regulated learning, thereby maximizing the educational value of generative AI.

## 2 Literature review

### 2.1 Technology acceptance model and the intention to use generative AI

The Technology Acceptance Model (TAM) has long been widely used to explain and predict users’ adoption behaviors of new technologies. The core logic of the model is that users’ intention to use a technology is primarily determined by its perceived ease of use and perceived usefulness (Davis, 1989; Davis et al., 1989). Perceived ease of use refers to the degree to which users perceive that using the technology requires minimal effort, while perceived usefulness reflects users’ belief in how much the technology enhances their work or learning performance. In the context of generative AI assisting second language writing learning, understanding learners’ intention to use these tools is a critical starting point for investigating how learners interact with AI and ultimately influence their writing outcomes (Liang et al., 2024; Zou et al., 2025).

In recent years, the rapid proliferation of generative AI, such as ChatGPT, has prompted scholars to apply TAM to investigate EFL learners’ acceptance of these writing assistance tools. Studies have shown that the perceived ease of use and usefulness of generative AI are key drivers of their adoption. When learners perceive generative AI as easy to use and capable of providing effective, personalized support in areas such as writing feedback, idea generation, language refinement, and text generation, thereby significantly enhancing writing efficiency and text quality, their intention to adopt these technologies is strengthened (Alharbi and Hassan Al-Ahdal, 2025; Shen et al., 2025).

In addition to the traditional dimensions of ease of use and usefulness, a key distinguishing feature of generative AI—compared to earlier educational technologies—is its high interactivity (Min and Li, 2025). While perceived ease of use concerns the effort required to operate the tool, perceived usefulness focuses on the instrumentality of the tool in improving writing outcomes, and perceived interactivity captures the extent to which learners experience dynamic, human-like, two-way communication with the AI during the writing process. Perceived interactivity (PI) refers to the degree to which learners perceive the ability to engage in real-time, dynamic, two-way communication with the AI (Kohnke et al., 2023). This anthropomorphic interaction capability fulfills learners’ social presence needs, thereby stimulating their interest and willingness to use the tools (Tai and Chen, 2023). Research indicates that generative AI with high interactivity not only makes the writing process more engaging but also sustains learners’ involvement through continuous dialogue and feedback cycles, positively influencing their intention to adopt these tools (Zhou and Hou, 2024; Zou et al., 2025).

In conclusion, in the context of generative AI-assisted second language writing learning, learners’ intention to use these tools is not solely influenced by a single factor. Instead, it is shaped by a combination of their perceptions of the tool’s usefulness, ease of use, and interactivity. Based on the TAM framework and existing empirical research, the following hypotheses are proposed:

*H1*: Perceived ease of use of generative AI positively influences EFL learners’ intention to use them.

*H2*: Perceived usefulness of generative AI positively influences EFL learners’ intention to use them.

*H3*: Perceived interactivity of generative AI positively influences EFL learners’ intention to use them.

### 2.2 Intention to use generative AI and learning engagement

Intention to use, as a key behavioral intention variable in TAM, serves as an important bridge between technology perceptions and actual behavior (Davis, 1989). In the context of generative AI-assisted second language learning, intention to use reflects learners’ psychological tendency to adopt and continuously use AI tools for learning, while learning engagement refers to the depth of cognitive, emotional, and behavioral involvement learners exhibit during their interaction with AI tools (Fredricks et al., 2004; Ma and Chen, 2024). Existing research has shown that intention to use is a key psychological factor that drives learners from “willingness to try” to “deep usage.” When learners hold a strong intention to use generative AI, they are more likely to integrate it into their daily learning processes, thereby increasing the frequency and intensity of high-involvement learning behaviors (Wang and Xue, 2024; Zhou and Hou, 2024). For instance, in AI-assisted writing tasks, a strong intention to use will prompt learners to frequently seek feedback from AI, invest more time revising drafts, and engage in deeper metacognitive reflection (Teng, 2025). This pattern is consistent with Chan et al. (2024), who observed heightened engagement and motivation among students receiving AI feedback. Therefore, the following hypothesis is proposed:

*H4*: The intention to use generative AI positively influences learning engagement.

### 2.3 Learning engagement and self-regulated learning

Learning engagement is a key precursor to academic achievement (Fredricks et al., 2004). In the context of generative AI-assisted second language learning, high levels of engagement are characterized by learners’ active use of AI tools for deep interaction, continuous exploration, and reflective practice (Guo and Wang, 2025; Zong and Yang, 2025). Self-regulated learning (SRL), on the other hand, emphasizes the use of metacognitive strategies by learners, including goal-setting, process monitoring, strategy adjustment, and reflection on outcomes (Zimmerman, 2002). Existing research suggests that learning engagement and self-regulated learning are dynamically interdependent (Shi L. et al., 2025). When learners are highly engaged, they are more likely to activate and effectively apply self-regulation strategies (Yang and Du, 2025). For example, in AI-assisted writing tasks, high levels of engagement often accompany more refined writing planning and process monitoring, thereby promoting the development of self-regulated learning (Hapsari and Rizky, 2025; Liu and Zhang, 2025). Lo et al. (2025) further underscore this relationship, demonstrating that engagement with feedback—whether from AI, teachers, or hybrid sources—is central to writing development, with students reporting varied motivational responses that influence their regulatory processes. Therefore, this study proposes the following hypothesis:

*H5*: Learning engagement positively influences self-regulated learning.

### 2.4 Intention to use generative AI and self-regulated learning

Intention to use, as a key motivational construct in the TAM, reflects learners’ willingness to adopt and persistently utilise generative AI for learning tasks (Davis, 1989). Within the context of AI-assisted EFL writing, this intention may play a crucial role beyond initial adoption, potentially shaping learners’ self-regulatory processes. When learners possess a strong intention to use AI tools, they are more likely to actively seek AI-generated feedback, set specific writing goals, and monitor their progress during task execution (Liu et al., 2024). Such goal-directed and persistent engagement with AI tools provides authentic opportunities for learners to practice and internalise self-regulated learning strategies, including planning, monitoring, and reflection (Teng, 2025). Furthermore, learners with higher usage intentions tend to view AI as a learning partner, which may enhance their metacognitive awareness and regulatory control during writing tasks (Behforouz and Al Ghaithi, 2025). Therefore, this study proposes the following hypothesis:

*H6*: The intention to use generative AI positively influences self-regulated learning.

### 2.5 Generative AI and self-regulated learning

The technical characteristics of generative AI may also directly shape learners’ SRL. Generative AI tools, such as ChatGPT, through their powerful natural language generation and contextualized interaction capabilities, provide learners with a highly personalized, real-time feedback environment that allows for autonomous exploration of learning content (Kohnke et al., 2023). This feature enables AI tools to surpass traditional learning aids by directly influencing learners’ core dimensions of SRL—goal setting, strategy use, and reflection (Behforouz and Al Ghaithi, 2025).

Specifically, the perceived ease of use (PEU) of generative AI may reduce the cognitive load associated with using the technology, allowing learners to allocate more cognitive resources to the learning process itself rather than to tool operation, thereby creating cognitive space for self-regulation strategies (Du, 2025). Research on AI speech evaluation systems has shown that the ease of use of tools significantly alleviates learners’ cognitive load, enabling them to focus more on practicing and adjusting speaking strategies (Zou et al., 2025). Additionally, Hou and Zhou (2025), based on SRL’s three-phase model, further highlight that user-friendly AI tools facilitate a smoother transition from “learning planning” to “strategy execution,” strengthening self-regulation during the performance phase.

Perceived usefulness (PU) of generative AI may also directly facilitate the development of learners’ self-regulated learning. When learners perceive that AI effectively enhance their learning efficiency and outcomes, they are more likely to set challenging learning goals and actively use cognitive and metacognitive strategies. This is because personalized paths provided by AI enhance learners’ sense of control over the learning process (Wei, 2023; Huang and Derakhshan, 2025). Similarly, Liu G. L. et al. (2025) found that the real-time feedback and content generation support provided by generative AI in writing tasks directly aid learners in developing more refined writing plans and process monitoring.

Of particular importance, the perceived interactivity (PI) of generative AI may stimulate learners’ motivation and reflective practice by creating human-like conversational scenarios. Highly interactive AI can simulate learning partners, guiding learners to engage in high-level cognitive activities such as explaining, questioning, and summarizing (Tai and Chen, 2023). For example, in AI-assisted writing tasks, dynamic, multi-turn conversations not only enhance task engagement but also prompt learners to continuously compare AI feedback with their own outputs, leading to deep reflection (Liu and Zhang, 2025). This “dialogical” interaction essentially externalizes metacognitive monitoring, directly promoting learners’ reflective regulation. Derakhshan (2025) suggests that GAI tools, by providing personalized, interactive support, directly meet learners’ autonomy needs, thereby enhancing their self-regulation motivation. Additionally, Yang and Du (2025) reveal that high-level self-regulated learners in AI-driven digital learning environments are characterized by their ability to effectively leverage the interactivity of AI for autonomous exploration. This suggests that perceived interactivity not only serves as a precursor to technology acceptance but may also directly empower learners’ self-regulatory practices.

Based on the above analysis, this study proposes the following hypotheses:

*H7*: Perceived ease of use of generative AI positively influences EFL learners’ self-regulated learning.

*H8*: Perceived usefulness of generative AI positively influences EFL learners’ self-regulated learning.

*H9*: Perceived interactivity of generative AI positively influences EFL learners’ self-regulated learning.

### 2.6 The relationship between generative AI, intention to use, learning engagement, and self-regulated learning

The impact of generative AI on learners’ self-regulated learning, within the integrated framework of TAM and SRL theory, does not follow a straightforward path. Instead, it represents a mediated chain process, influenced by multiple cognitive, behavioral, and emotional factors (Zimmerman, 2002; Hou and Zhou, 2025). As discussed in Sections 2.1–2.4, the perceived characteristics of generative AI (ease of use, usefulness, and interactivity) initially stimulate intention to use (H1–H3). This intention, in turn, fosters learners’ deep engagement across cognitive, emotional, and behavioral dimensions (H4), with learning engagement serving as a critical mediator that directly and positively influences the development of self-regulated learning (H5). Therefore, it is crucial to further elucidate the complete pathway of “technology perceptions → intention to use → behavioral engagement → regulatory processes” through the lens of chain mediation.

Specifically, the perceived ease of use (PEU) of generative AI influences self-regulated learning through the aforementioned chain process. When learners perceive AI tools as user-friendly and seamless to interact with, their cognitive load is reduced, and resistance to technology adoption is minimized (Zou et al., 2025), thereby facilitating the formation of a positive intention to use. A strong intention to use further translates into sustained learning behaviors and emotional engagement. As noted by Tai and Chen (2024), user-friendly generative AI voice assistants significantly enhance students’ classroom participation and learning engagement. Through the mediating effect of learning engagement, learners redirect the cognitive resources saved toward actively regulating the learning process, such as setting short-term goals and adjusting learning strategies based on AI feedback (Liu and Zhang, 2025).

Similarly, the perceived usefulness (PU) of generative AI indirectly influences self-regulated learning by fostering intention to use and enhancing learning engagement. When learners believe that AI tools can effectively improve their language performance, their intention to adopt these technologies increases significantly (Liu and Ma, 2024). A strong intention to use encourages learners to engage more frequently and meaningfully with AI tools for personalized practice and reflection, thereby enhancing their behavioral focus, cognitive strategies, and emotional involvement in learning tasks (Wang and Xue, 2024; Yuan and Liu, 2025). This multidimensional engagement provides the essential foundation for applying self-regulation strategies.

Crucially, the perceived interactivity (PI) of generative AI strengthens the chain of influence from intention to use to learning engagement, ultimately facilitating self-regulated learning. Interactive AI tools simulate real-time conversations, providing contextualized and dynamic responses that enhance learners’ experiences (Kohnke et al., 2023). This feature not only influences intention to use (as indicated in H3) but also fosters deeper engagement by making the learning process more immersive and cultivating a sense of social presence (Zhou and Hou, 2024). It is clear that interactivity, through continuous mediation of the “intention to use → engagement” pathway, indirectly promotes learners’ self-regulated learning.

In summary, intention to use and learning engagement play an mediating roles in the relationship between the perceived characteristics of generative AI and self-regulated learning. This pathway reflects a progressive mechanism of “technology acceptance → behavioral immersion → regulatory process,“which aligns with Zimmerman’s (2002) social cognitive view of self-regulated learning, wherein environmental factors, such as technological tools, influence individual behaviors and psychological processes, and ultimately fosters the development of self-regulated learning. Therefore, the following hypotheses are proposed:

*H10*: The perceived ease of use of generative AI indirectly influences learners’ self-regulated learning through intention to use.

*H11*: The perceived ease of use of generative AI indirectly influences learners’ self-regulated learning through intention to use and learning engagement.

*H12*: The perceived usefulness of generative AI indirectly influences learners’ self-regulated learning through intention to use.

*H13*: The perceived usefulness of generative AI indirectly influences learners’ self-regulated learning through intention to use and learning engagement.

*H14*: The perceived interactivity of generative AI indirectly influences learners’ self-regulated learning through intention to use.

*H15*: The perceived interactivity of generative AI indirectly influences learners’ self-regulated learning through intention to use and learning engagement.

### 2.7 Research model

**Figure 1:**
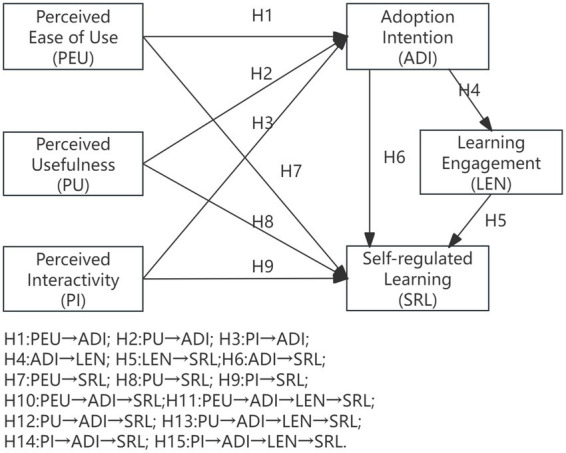
Conceptual framework.

Integrating TAM and SRL, this study constructs a conceptual framework of “perceived technology characteristics →behavioral intention→immersive engagement → regulatory process” to explore the impact and mechanisms through which generative AI assists second language learning and influence learners’ self-regulated learning. The model treats the perceived usefulness, ease of use, and interactivity of generative AI as independent variables, with intention to use and learning engagement serving as mediators, and self-regulated learning as the dependent variable (see Figure 1).

## 3 Research methodology

### 3.1 Sampling and sample

This study surveyed EFL students from several universities in East China. The sample selection focused on this group for two main reasons: first, Chinese EFL learners generally face dual challenges in academic writing, namely language expression and logical structuring. Generative AI, by providing instant feedback and content support, may significantly influence their learning behaviors and the application of self-regulated learning strategies. Second, this group shows a relatively high acceptance of AI technologies, which facilitates a deeper exploration of the learning mechanisms under technological intervention. Data collection was conducted from September to December 2025 through an online questionnaire survey, with a snowball sampling method used to expand the sample coverage. To reduce sampling bias, initial seeds were diversified across institutions, and respondents were encouraged to forward the survey widely. To ensure data quality, several screening protocols were implemented: duplicate responses were identified and removed based on IP addresses; responses with completion time below 120 s were excluded; and straightlining/longstring patterns (e.g., selecting the same option for all items) were detected and excluded. After this rigorous data cleaning process, a total of 386 valid responses were collected, meeting the sample size requirements for structural equation modeling analysis. Participants were aged between 18 and 23 years, with 69.17% (*n* = 267) female and 30.83% (*n* = 119) male, reflecting the typical gender distribution in the field of foreign language learning. In terms of AI tool usage frequency, 31.35% (*n* = 121) reported using AI tools daily, 45.60% (*n* = 176) used them several times a week, and 23.06% (*n* = 89) used them a few times a month, indicating that AI tools have become deeply integrated into their learning processes. The distribution of AI tool types showed that DeepSeek (30.31%) and ChatGPT (24.61%) were the most commonly used platforms, representing a range of generative AI both domestically and internationally, thereby enhancing the representativeness of the sample. Overall, the sample exhibited good diversity in terms of demographic characteristics and AI usage patterns.

### 3.2 Instruments

The questionnaire used in this study consists of two parts: the first part collected demographic information, while the second part included measurement items for the core constructs. All items were measured using a five-point Likert scale (from “Strongly Disagree” to “Strongly Agree”). The measurement items for each construct were primarily adapted from established scales and modified to fit the specific context of generative AI-assisted second language writing, and expert review and pilot testing were conducted to ensure reliability and validity (See Supplementary Tables 1–5).

#### 3.2.1 Perceived ease of use

The assessment of perceived ease of use aims to evaluate learners’ perceptions of how easy it is to use generative AI in assisting with second language writing. This study adapted scales developed by Fan and Wang (2023), Liu and Ma (2024), and Hu and Gong (2025), including five items, such as: “It is easy for me to learn how to use generative AI to assist with second language writing.”

#### 3.2.2 Perceived usefulness

The measurement of perceived usefulness aims to assess learners’ perceptions of how generative AI enhance their second language writing performance. Drawing on the work of Fan and Wang (2023), Liu and Ma (2024), and Hu and Gong (2025), five items were designed, such as: “Using generative AI is beneficial to the quality of my second language writing.”

#### 3.2.3 Perceived interactivity

Perceived interactivity measures learners’ perceptions of the degree to which they engage in bidirectional, dynamic interactions with generative AI. Based on scales developed by Etemad-Sajadi (2016) and Pillai et al. (2024), five items were designed, such as: “Generative AI allows me to interact with them in a conversational manner to receive the writing support I need.”

#### 3.2.4 Intention to use

Intention to use refers to learners’ tendency to adopt and consistently use generative AI. The measurement items were adapted from the scales developed by Tarhini et al. (2017) and Pillai et al. (2024), consisting of five items, such as: “Whenever I have a second language writing task, I try to use generative AI to assist me.”

#### 3.2.5 Learning engagement

Learning engagement measures the degree of learners’ concentration and involvement in the AI-assisted writing process. The items were adapted from Schaufeli et al.’s (2019) work engagement scale and were further refined based on applications in educational contexts by Hey et al. (2024) and Liu M. et al. (2025), consisting of three items, such as: “When using generative AI for writing, I feel fully engaged and energized.”

#### 3.2.6 Self-regulated learning

Self-regulated learning is assessed through learners’ behaviors in setting goals, monitoring progress, and reflecting on outcomes during AI-assisted writing. The items were developed based on the classic framework by Zimmerman and Pons (1986), with reference to Shi J. et al. (2025), and include three items, such as: “When using generative AI, I set clear goals for my writing tasks.”

It should be noted that learning engagement and self-regulated learning were operationalized using brief three-item scales capturing global perceptions of these constructs. These measures were intended to serve as concise unidimensional indicators rather than comprehensive multidimensional assessments.

### 3.3 Data analysis method

Data analysis proceeded in several stages using SPSS 26.0 and SmartPLS 3.0. First, descriptive statistics were computed to understand the data distribution. The measurement model was then assessed for reliability, convergent validity (using CA, CR, AVE), and discriminant validity (via the Fornell–Larcker criterion and HTMT). Subsequently, structural equation modeling (SEM) was employed to test the hypothesized paths and the chain mediation effects, with significance determined by bootstrapping. To account for potential individual differences and enhance the precision of the model, learners’ gender, the specific AI tool they primarily use, and their frequency of AI usage were included as control variables in the analysis. To ensure robustness, we further conducted multicollinearity tests (VIF), common method bias analyses with CFA marker-variable approach, multi-group analyses (gender, AI usage frequency), and comparisons with alternative theoretical models.

## 4 Results

### 4.1 Descriptive analysis

The descriptive statistical analysis results (see Table 1) indicate that the mean values of the key variables ranged from 3.47 to 3.76, suggesting that participants’ perceptions of AI tools and their overall learning states were generally at a moderately high level. Specifically, participants reported higher scores for the perceived interactivity of generative AI (*M* = 3.71, SD = 0.91), perceived ease of use (*M* = 3.66, SD = 0.91), and perceived usefulness (*M* = 3.56, SD = 0.96), reflecting the strengths of current AI tools in terms of interactivity, ease of use, and usefulness. Additionally, intention to use (*M* = 3.76, SD = 0.90) was relatively high, indicating that learners were generally willing to engage in AI-assisted learning activities. In contrast, learning engagement had a slightly lower mean (*M* = 3.61, SD = 0.90), and self-regulated learning had the lowest mean (*M* = 3.47, SD = 0.92), suggesting that respondents exhibited some degree of learning engagement and self-regulated learning activities, albeit at relatively lower levels.

**Table 1:** Descriptive statistics.

Construct	Minimum	Maximum	Mean	Standard deviation	Skewness	Kurtosis
PEU	1.00	5.00	3.66	0.91	−0.63	−0.18
PU	1.00	5.00	3.56	0.96	−0.72	−0.26
PI	1.00	5.00	3.71	0.91	−0.73	0.09
ADI	1.20	5.00	3.76	0.90	−0.68	−0.09
LEN	1.00	5.00	3.61	0.90	−0.48	−0.01
SRL	1.00	5.00	3.47	0.92	−0.66	0.23

### 4.2 Reliability and validity

The reliability and convergent validity of the constructs were assessed using three indicators: composite reliability, Cronbach’s *α* coefficient, and average variance extracted (Hair et al., 2010). As shown in Table 2, the composite reliability for all six constructs was greater than 0.80, demonstrating good reliability (Anderson and Gerbing, 1988). Cronbach’s α coefficients also exceeded 0.80, further supporting the internal consistency of the constructs (Fornell and Larcker, 1981). Additionally, the average variance extracted (AVE) for all constructs exceeded 0.60, confirming adequate convergent validity (Hair et al., 2010).

**Table 2:** Reliability and convergent validity.

Construct	CA	CR	AVE
ADI	0.937	0.952	0.800
LEN	0.871	0.921	0.795
PEU	0.921	0.940	0.760
PI	0.914	0.935	0.743
PU	0.930	0.947	0.781
SRL	0.862	0.916	0.784

CA, Cronbach’s alpha; CR, Composite Reliability; AVE, Average Variance Extracted.

Discriminant validity was assessed by Fornell–Larcker criterion and heterotrait–monotrait ratio (HTMT). According to the rule of thumb, a construct is considered to have acceptable discriminant validity if the square root of its AVE exceeds the highest correlation with any other construct (Chin, 1998). As shown in Table 3, the square roots of the AVE (presented in parentheses) are greater than the corresponding off-diagonal correlations, indicating that these constructs exhibit acceptable discriminant validity. As shown in Table 4, All HTMT values were below the recommended threshold of 0.85/0.90, supporting adequate discriminant validity among the constructs.

**Table 3:** Fornell–Larcker criterion.

Fournier- lackel values	ADI	LEN	PEU	PI	PU	SRL
ADI	(0.895)					
LEN	0.562	(0.892)				
PEU	0.620	0.542	(0.872)			
PI	0.577	0.610	0.644	(0.862)		
PU	0.557	0.497	0.570	0.606	(0.883)	
SRL	0.574	0.555	0.556	0.580	0.596	(0.883)

**Table 4:** Heterotrait–Monotrait ratio (HTMT).

HTMT values	ADI	LEN	PEU	PI	PU	SRL
ADI						
LEN	0.616					
PEU	0.664	0.603				
PI	0.619	0.683	0.700			
PU	0.592	0.550	0.616	0.655		
SRL	0.634	0.639	0.624	0.651	0.663	

### 4.3 Multicollinearity test

The six construct extracted for this study were analyzed for collinearity. The results (see Table 5) indicate that all variables had VIF values lower than 3.3. According to Kock’s (2015) perspective, a VIF > 3.3 is considered indicative of pathological multicollinearity, which could be affected by common method bias and contamination. This finding suggests that the measurements in this study are not significantly affected by covariance issues.

**Table 5:** VIF of latent variables.

VIF values	ADI	LEN	PEU	PI	PU	SRL
ADI		1.000				2.011
LEN						1.845
PEU	1.873					2.153
PI	1.996					2.293
PU	1.732					1.870
SRL						

### 4.4 Model acceptance and interpretability

Explained variance (*R*^2^): The model accounted for a substantial proportion of variance in the endogenous constructs. Specifically, as shown in Table 6, the predictors explained *R*^2^ = 0.468 of the variance in intention to use (ADI), *R*^2^ = 0.315 in learning engagement (LEN), and *R*^2^ = 0.501 in self-regulated learning (SRL), indicating moderate explanatory power. Moreover, the model’s SRMR (Standardized Root Mean Square Residual) is 0.07 (with SRMR < 0.08 indicating an acceptable model fit), and the NFI (Normed Fit Index) is 0.887 (with NFI closerto 1 indicating a better fit). Therefore, the structural equation model constructed in this study still possesses a certain level of explanatory power and persuasiveness.

**Table 6:** Model acceptance and interpretability.

*R* ^2^			SRMR	NFI
ADI	LEN	SRL		
0.468	0.315	0.501	0.070	0.887

### 4.5 Hypothesis test

**Figure 2:**
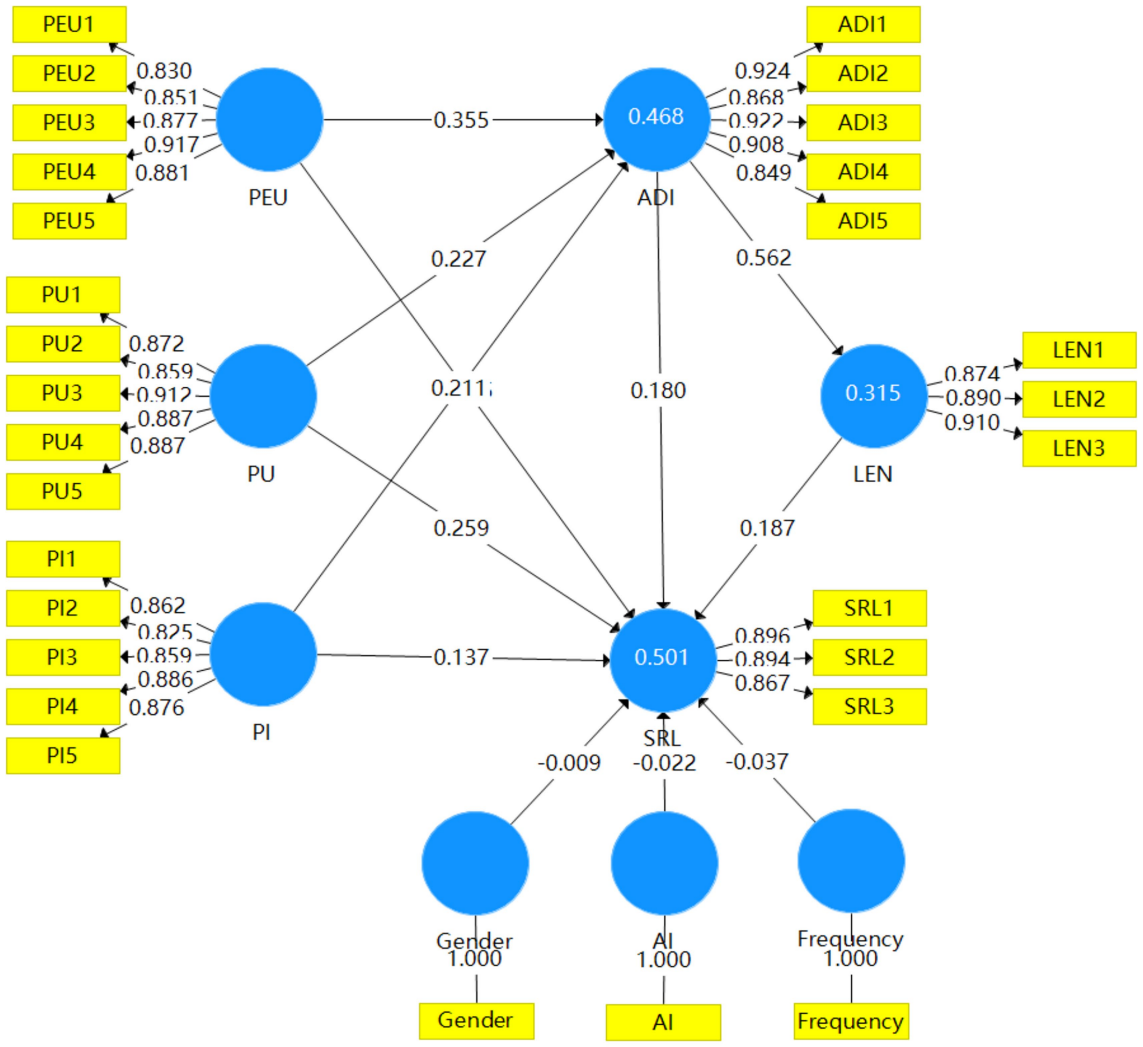
Structural model.

To test the theoretical hypotheses proposed in this study, structural equation modeling (SEM) was employed to estimate the path coefficients and their significance levels. The analysis results, presented in Table 7 and Figure 2, provide comprehensive empirical evidence for the hypothesized relationships among the constructs.

**Table 7:** Direct and indirect effects.

Path	Original sample (*O*)	Sample mean (*M*)	Confidence interval		STDEV	Significance	
			2.5%	97.5%		*T*	*P*
Total effects							
ADI → LEN	0.562	0.562	0.492	0.624	0.034	16.728	0.000
ADI → SRL	0.286	0.283	0.165	0.394	0.058	4.931	0.000
AI → SRL	−0.022	−0.023	−0.093	0.047	0.036	0.624	0.533
Frequency → SRL	−0.037	−0.036	−0.110	0.037	0.038	0.973	0.331
Gender → SRL	−0.009	−0.009	−0.081	0.064	0.037	0.238	0.812
LEN → SRL	0.187	0.185	0.099	0.271	0.044	4.295	0.000
PEU → ADI	0.355	0.354	0.247	0.462	0.055	6.502	0.000
PEU → LEN	0.199	0.199	0.135	0.265	0.033	5.989	0.000
PEU → SRL	0.207	0.206	0.103	0.306	0.051	4.025	0.000
PI → ADI	0.211	0.210	0.113	0.302	0.048	4.386	0.000
PI → LEN	0.118	0.118	0.062	0.174	0.029	4.138	0.000
PI → SRL	0.197	0.198	0.079	0.310	0.060	3.295	0.001
PU → ADI	0.227	0.229	0.122	0.337	0.055	4.160	0.000
PU → LEN	0.128	0.129	0.068	0.195	0.032	3.956	0.000
PU → SRL	0.324	0.325	0.227	0.421	0.048	6.694	0.000
Direct effects							
ADI → LEN	0.562	0.562	0.492	0.624	0.034	16.728	0.000
ADI → SRL	0.180	0.180	0.068	0.284	0.056	3.240	0.001
AI → SRL	−0.022	−0.023	−0.093	0.047	0.036	0.624	0.533
Frequency → SRL	−0.037	−0.036	−0.110	0.037	0.038	0.973	0.331
Gender → SRL	−0.009	−0.009	−0.081	0.064	0.037	0.238	0.812
LEN → SRL	0.187	0.185	0.099	0.271	0.044	4.295	0.000
PEU → ADI	0.355	0.354	0.247	0.462	0.055	6.502	0.000
PEU → SRL	0.105	0.106	0.004	0.205	0.052	2.046	0.041
PI → ADI	0.211	0.210	0.113	0.302	0.048	4.386	0.000
PI → SRL	0.137	0.138	0.013	0.258	0.063	2.177	0.030
PU → ADI	0.227	0.229	0.122	0.337	0.055	4.160	0.000
PU → SRL	0.259	0.260	0.165	0.354	0.048	5.408	0.000
Indirect effects							
PEU → ADI → LEN	0.199	0.199	0.135	0.265	0.033	5.989	0.000
PI → ADI → LEN	0.118	0.118	0.062	0.174	0.029	4.138	0.000
PU → ADI → LEN	0.128	0.129	0.068	0.195	0.032	3.956	0.000
PEU → ADI → SRL	0.064	0.064	0.022	0.111	0.023	2.785	0.005
PI → ADI → SRL	0.038	0.038	0.012	0.068	0.014	2.638	0.008
PU → ADI → SRL	0.041	0.041	0.013	0.076	0.016	2.511	0.012
PEU → ADI → LEN → SRL	0.037	0.037	0.018	0.059	0.011	3.513	0.000
PI → ADI → LEN → SRL	0.022	0.022	0.009	0.038	0.007	3.097	0.002
ADI → LEN → SRL	0.105	0.104	0.055	0.155	0.025	4.169	0.000
PU → ADI → LEN → SRL	0.024	0.024	0.010	0.043	0.009	2.794	0.005

Regarding the technology acceptance path, the perceived characteristics of generative AI significantly and positively predicted learners’ intention to use. Specifically, perceived ease of use (*β* = 0.355, *p* < 0.001), perceived usefulness (*β* = 0.227, *p* < 0.001), and perceived interactivity (*β* = 0.211, *p* < 0.001) all exerted significant positive effects on intention to use, thereby supporting hypotheses H1, H2, and H3. These findings align with the core propositions of the Technology Acceptance Model (Davis, 1989) and extend its applicability to the context of generative AI-assisted EFL writing.

For the learning engagement pathway, intention to use demonstrated a strong positive influence on learning engagement (*β* = 0.562, *p* < 0.001), confirming hypothesis H4. This result indicates that learners’ willingness to adopt AI tools effectively translates into deeper cognitive, emotional, and behavioral involvement in writing tasks. Furthermore, learning engagement significantly and positively predicted self-regulated learning (*β* = 0.187, *p* < 0.001), supporting hypothesis H5 and underscoring the crucial role of engagement as a precursor to the activation of self-regulatory strategies (Fredricks et al., 2004).

Regarding the direct effects on self-regulated learning, both the perceived characteristics of generative AI and intention to use showed significant positive relationships. Intention to use exerted a significant direct effect on self-regulated learning (*β* = 0.180, *p* < 0.01), thereby supporting hypothesis H6. This finding suggests that beyond its indirect influence through engagement, learners’ willingness to adopt AI tools directly contributes to the activation and application of self-regulated learning strategies during writing tasks. Among the technology perception variables, perceived usefulness exerted the strongest direct effect (*β* = 0.259, *p* < 0.001), followed by perceived interactivity (*β* = 0.137, *p* < 0.05) and perceived ease of use (*β* = 0.105, *p* < 0.05), thus supporting hypotheses H7, H8, and H9.

Notably, the bootstrapping analysis also confirmed the mediating roles of intention to use and learning engagement. The indirect effects of perceived ease of use (*β* = 0.064, *p* < 0.01), perceived usefulness (*β* = 0.041, *p* < 0.05), and perceived interactivity (*β* = 0.038, *p* < 0.01) on self-regulated learning through intention to use were all significant, supporting hypotheses H10, H12, and H14. More importantly, the chain mediation effects of “intention to use → learning engagement” were also significant for perceived ease of use (*β* = 0.037, *p* < 0.001), perceived usefulness (*β* = 0.024, *p* < 0.01), and perceived interactivity (*β* = 0.022, *p* < 0.01), thereby confirming hypotheses H11, H13, and H15. These results reveal the sequential psychological process through which technology perceptions influence self-regulated learning.

Finally, the control variables—gender, specific AI tool type, and frequency of AI usage—did not exhibit significant effects on self-regulated learning, suggesting that the proposed model maintains its explanatory power across different demographic and usage contexts.

### 4.6 Multi-group test

To examine whether the proposed relationships varied across different demographic and usage contexts, multi-group analyses were conducted for gender and AI usage frequency. The results indicate no significant differences were found between male and female learners across all paths (*p* > 0.05), suggesting that gender does not moderate the structural relationships in the model. Regarding AI usage frequency (daily, weekly, or monthly users), the results in Supplementary Table 3 indicate that the majority of path coefficients were not significantly different across groups. However, a notable exception was observed for the direct effect of perceived usefulness on self-regulated learning (PU → SRL), which differed significantly between daily and monthly users (*p* < 0.05), suggesting that frequent users may derive greater self-regulatory benefits from perceiving AI as useful. Overall, these findings demonstrate that the proposed chain mediation model is largely robust across genders and usage frequencies. The multi-group analysis was conducted as an exploratory robustness check to examine potential differences in structural relationships across gender and AI usage frequency groups. A formal measurement invariance procedure (e.g., MICOM) was not implemented; therefore, these comparisons should be interpreted as indicative rather than confirmatory tests of invariance (see Supplementary Tables 6, 7).

### 4.7 Common method bias test with CFA marker-variable approach

To test for common method bias, a CFA marker-variable approach was employed. The marker variable selected was the Brief Social Desirability Scale (BSDS), developed by Haghighat (2007). The BSDS is a 4-item scale designed to measure an individual’s tendency to respond in a socially desirable manner. Its four items capture a general response style related to self-presentation, which is conceptually distinct from the focal constructs (PEU, PU, PI, ADI, LEN, SRL) investigated in our model. Therefore, it served as an ideal marker, as any significant correlation between it and the substantive constructs would suggest the influence of common method bias.

To empirically verify this theoretical irrelevance, the correlations between the BSDS and all other latent constructs were examined. The correlations between the BSDS marker variable and the focal constructs were all very low and non-significant (ranging from −0.081 to −0.006, all *p* > 0.05). Furthermore, the results indicate all paths from the marker variables to the latent constructs were non-significant (*p* > 0.05). Furthermore, after including these marker variables, the path coefficients and significance levels of the main hypothesized relationships remained substantially unchanged. These results indicate that common method bias does not pose a serious concern in this study (see Supplementary Tables 8, 9).

### 4.8 Comparisons with alternatives model

To further validate the superiority of the proposed chain mediation model, two alternative models were tested and compared. Alternative Model 1, which omitted the direct path from intention to use to self-regulated learning (ADI → SRL), exhibited a lower explained variance in SRL (*R*^2^ = 0.478) and a higher SRMR (0.078) than the proposed model. Alternative Model 2, which reversed the order by positioning learning engagement as an antecedent to intention to use (LEN → ADI), yielded the poorest fit (SRMR = 0.110). These comparisons confirm that the proposed model, with its full mediation chain and theoretically grounded directional paths, offers the best explanatory power and model fit (see Supplementary Table 10).

## 5 Discussion

### 5.1 Key findings and analysis

The structural equation modeling analysis yielded several noteworthy findings that elucidate the mechanisms through which generative AI influences EFL learners’ self-regulated learning. First, consistent with the Technology Acceptance Model (Davis, 1989), learners’ perceptions of generative AI—specifically its ease of use, usefulness, and interactivity—collectively shaped their willingness to adopt these tools for writing tasks. This confirms that traditional TAM constructs remain relevant in the context of generative AI, while the addition of perceived interactivity—a distinctive affordance of conversational AI—extends the model’s explanatory power in technology-rich language learning environments.

Second, the hypothesized chain mediation pathway received strong empirical support. The findings reveal that learners’ intention to use AI tools serves as a critical bridge, translating positive technology perceptions into deeper learning engagement. When learners develop strong usage intentions, they are more likely to invest cognitive, emotional, and behavioral energy in AI-assisted writing tasks. This immersive engagement, in turn, provides the fertile ground for self-regulated learning to flourish, as actively engaged learners naturally exercise goal-setting, progress monitoring, and reflective strategies. This sequential pattern validates the theoretical framework that positions technology acceptance not as an endpoint, but as a gateway to deeper psychological processes.

Third, among the technology perception variables, perceived usefulness emerged as the most potent predictor of self-regulated learning. This finding aligns with the motivational role of perceived task value in metacognitive regulation: when learners genuinely believe that AI enhances their writing performance, they are more inclined to set challenging goals, monitor their progress, and reflect on outcomes (Huang and Derakhshan, 2025). The direct contributions of perceived ease of use and perceived interactivity, though comparatively modest, remained meaningful, suggesting that cognitive accessibility and interactive dialogue each independently support self-regulatory development.

Fourth, the analysis revealed that intention to use exerts a direct influence on self-regulated learning, even after accounting for the mediating role of engagement. This suggests that the willingness to adopt AI tools can independently activate self-regulatory strategies—perhaps by prompting learners to mentally plan how to leverage the tool, anticipate its feedback, and adjust their writing approaches. This pattern aligns with partial mediation logic (Liu et al., 2024) and underscores that motivational states can trigger metacognitive processes in their own right.

Comparisons with prior studies enrich the interpretation of these findings. While earlier research has documented that ChatGPT use is associated with self-regulation among EFL writers (Liu et al., 2024), such work has typically not unpacked the underlying mechanisms. The present study extends this literature by revealing the sequential psychological pathway—from technology perceptions through intention and engagement to self-regulation—that explains how AI tools translate into higher-order learning outcomes. This pathway aligns with the evidence provided by Chan et al. (2024), whose randomized controlled trial demonstrated that AI feedback significantly improved essay quality, engagement, and motivation. Similarly, the prominent role of perceived interactivity is corroborated by Lo et al. (2025), who found that students valued the personalized, interactive nature of human feedback over AI-only feedback, underscoring the importance of dialogic interaction in fostering engagement and, ultimately, self-regulated learning.

Overall, these findings advance the integration of TAM and SRL theory by demonstrating that generative AI enhances self-regulated learning through a progressive chain of motivational, behavioral, and regulatory processes. They highlight the importance of designing AI tools that are not only easy and useful but also highly interactive, and of crafting learning tasks that leverage these perceptions to foster sustained engagement and self-regulation.

### 5.2 Contributions

This study makes contributions to theory, practice, and methodology by constructing and validating a chain mediation model that systematically reveals how the perceived characteristics of generative AI influence EFL learners’ self-regulated learning through intention to use and learning engagement.

In terms of theoretical contributions, this study goes beyond the traditional limitations of the TAM, which focuses solely on adoption intention, by integrating it with learning engagement and self-regulated learning in educational contexts. It proposes an integrated theoretical framework of “technology perception → behavioral intention → immersive engagement → regulatory process.” This framework not only confirms the direct effects of perceived ease of use, perceived usefulness, and perceived interactivity on self-regulated learning but also illustrates how the “intention to use → engagement” pathway indirectly influences self-regulated learning. This deepens the understanding of the psychological mechanisms underlying AI-enhanced learning and offers a new theoretical perspective on second language writing in AI-powered learning environments.

In terms of practical contributions, this study provides clear guidance for educators and technology developers. The findings suggest that to maximize the educational benefits of AI, it is crucial to optimize the tools’ ease of use, usefulness, and interactivity in tandem. This optimization can effectively guide learners from “willing to use” to “deeply engaged,” ultimately achieving the ultimate goal of “learning effectively.” The study highlights the importance of fostering positive perceptions of AI tools among learners and designing highly interactive, engaging learning tasks that stimulate deep involvement and promote the development of self-regulated learning.

The methodological contribution of this study lies in its innovative use of structural equation modeling to empirically test the chain mediation effects of “intention to use” and “learning engagement” in the relationship between generative AI and self-regulated learning. This multiple mediation model unveils the complex, stepwise process from technology acceptance to higher-order skill development, offering greater complexity and explanatory power than a simple direct effect or parallel mediation model. It provides a new methodological framework for future research on technology-enabled learning.

## 6 Conclusions and future research

### 6.1 Conclusion

This study developed and validated a chain mediation model, which empirically examined how the perceived characteristics of generative AI (ease of use, usefulness, and interactivity) influence self-regulated learning in EFL learners. The key findings are as follows: First, the study confirms that the perceived ease of use, perceived usefulness, and perceived interactivity of generative AI significantly and positively predict learners’ intention to use AI, supporting the applicability of the TAM in this context. Second, the structural equation modeling (SEM) analysis suggests a theoretically specified sequential relationship: the perceived characteristics of AI tools not only have a significant direct positive effect on learners’ self-regulated learning (with perceived usefulness having the strongest direct effect), but they also exert an indirect influence on self-regulated learning through the “intention to use →learning engagement” pathway. When learners perceive AI tools as easy to use, genuinely useful, and highly interactive, they are more likely to adopt them. This strong intention to use, in turn, positively relates to deeper cognitive, emotional, and behavioral engagement in writing tasks, and sustained high-level engagement provides the necessary context and motivation for the application and internalization of self-regulation strategies. Based on these findings, several concrete pedagogical and design implications emerge. For educators, we recommend designing writing tasks that require multiple rounds of interaction with AI—such as iterative drafting, feedback-seeking, and revision cycles—to sustain engagement and foster self-regulation. For technology developers, enhancing AI functions that provide explicit scaffolding for writing strategies—for instance, prompting learners to set goals, offering metacognitive questions, and visualizing revision progress—could further support learners’ regulatory development. These actionable recommendations provide practical guidance for maximizing the educational potential of generative AI in language learning contexts.

### 6.2 Limitations and future research

While this study provides insights into the mechanisms by which generative AI affect self-regulated learning in EFL learners through the development and validation of a chain mediation model, several limitations remain, which also suggest directions for future research. First, the cross-sectional design and self-reported questionnaires used in this study reveal correlations between variables, but they do not establish strict causal relationships. Second, the sample in this study was composed of EFL learners from Chinese universities, which, while regionally representative, may limit the generalizability of the findings. Although the overrepresentation of female participants mirrors the typical gender distribution in foreign language education, it may still introduce gender bias. Third, the learning engagement scale employed in this study comprised only three items, adapted from the UWES-3. While its reliability was acceptable, the brevity of the scale may not fully capture the multidimensional nature of engagement. Fourth, this study mainly examined learners’ subjective perceptions of generative AI and did not explore the actual usage behaviors and their impact on the learning process. Future research can adopt longitudinal/experimental designs, expand samples across cultures and proficiencies, incorporate fine-grained behavioral indicators like prompt logs and revision trajectories to track human-AI interaction, and employ more comprehensive scales to measure learning engagement for nuanced findings.

## Data Availability

The original contributions presented in the study are included in the article/Supplementary material, further inquiries can be directed to the corresponding author.
