# Supplementary material for: How generative AI enhances self-regulated learning in EFL learners: a chain mediation model of “intention to use” and “learning engagement”
Source: Front Psychol. 2026 Apr 1;17:1808183. doi: 10.3389/fpsyg.2026.1808183 (PMC13079642; doi:10.3389/fpsyg.2026.1808183)
Supplement: Supplementary file 1 [file Supplementary_file_1.DOCX]

Supplementary Material

# Supplementary Table

Supplementary Table 1. Specific Assessment Items for Each Construct

| **Construct** | **Item No.** | **Item** | **Source** |
| --- | --- | --- | --- |
| **Perceived Ease of Use** | PEU1 | Using generative AI to assist in second language writing is easy for me. | Fan and Wang (2023); Liu and Ma (2024); Hu and Gong (2025) |
|  | PEU2 | The interaction process with generative AI in writing tasks is clear and easy to understand. |  |
|  | PEU3 | I find the interface of generative AI flexible and adaptable to my writing needs. |  |
|  | PEU4 | I think it is very easy to use generative AI to assist in second language writing. |  |
|  | PEU5 | Using generative AI for writing assistance requires little mental effort. |  |
| **Perceived Usefulness** | PU1 | Using generative AI helps improve the quality of my second language writing. | Fan and Wang (2023); Liu and Ma (2024); Hu and Gong (2025) |
|  | PU2 | Using generative AI helps me organize ideas and structure my articles more effectively. |  |
|  | PU3 | Using generative AI enhances my efficiency in completing second language writing tasks. |  |
|  | PU4 | Using generative AI increases my second language writing learning efficiency. |  |
|  | PU5 | Overall, I believe generative AI is useful for my second language writing development. |  |
| **Perceived Interactivity** | PI1 | Generative AI allows me to interact with it in a conversational manner to receive writing support. | Etemad-Sajadi (2016); Pillai et al. (2024) |
|  | PI2 | The interactive features of generative AI (such as multi-turn dialogue) effectively meet my writing needs. |  |
|  | PI3 | Generative AI helps me obtain the information or feedback I need instantly, without waiting for human assistance. |  |
|  | PI4 | I can obtain contextualized and personalized writing suggestions from generative AI through interactive communication. |  |
|  | PI5 | Generative AI interacts effectively with me to meet my writing support needs. |  |
| **Adoption Intention (ADI)** | ADI1 | Whenever I have a second language writing task, I will try to use generative AI to assist me. | Tarhini et al. (2017); Pillai et al. (2024) |
|  | ADI2 | I plan to use generative AI regularly to assist in my second language writing. |  |
|  | ADI3 | I plan to continue using generative AI as an important part of my second language learning process. |  |
|  | ADI4 | I would recommend my classmates use generative AI for second language writing assistance. |  |
|  | ADI5 | I am willing to invest time in learning how to use generative AI more effectively for writing. |  |
| **Learning Engagement** | LEN1 | When I use generative AI for writing, I feel fully engaged and energized. | Schaufeli et al. (2019); Hey et al. (2024); Liu et al. (2025a) |
|  | LEN2 | I am passionate about using generative AI to explore and improve my writing. |  |
|  | LEN3 | I often become deeply immersed in the writing process when interacting with generative AI. |  |
| **Self-regulated Learning** | SRL1 | When using generative AI, I set clear goals for my writing tasks. | Zimmerman and Pons (1986); Shi et al. (2025a) |
|  | SRL2 | I monitor my writing progress and adjust strategies based on feedback from generative AI. |  |
|  | SRL3 | I reflect on the suggestions provided by generative AI to improve my future writing. |  |

Supplementary Table 2. Content Validity (Expert Review)

To assess the content validity of the scale, five experts in the relevant field were invited to evaluate each item. The results showed that the **Item‑Content Validity Index (I‑CVI)**​ for all items ranged from 0.80 to 1.00, exceeding the recommended threshold of 0.78. Moreover, the **Scale‑Content Validity Index (S‑CVI)**​ was calculated as 0.96, which surpasses the commonly accepted criterion of 0.90. These findings indicate that the scale exhibits excellent content validity.

| Item No. | Expert evaluation | | | | | The total number of experts selected is 4 or 5 | Minimum | Maximum | Mean | Standard Deviation | n-A | I-CVI |
| --- | --- | --- | --- | --- | --- | --- | --- | --- | --- | --- | --- | --- |
|  | Expert1 | Expert2 | Expert3 | Expert4 | Expert5 |  |  |  |  |  |  |  |
| PEU1 | 5 | 5 | 5 | 5 | 4 | 5 | 4.00 | 5.00 | 4.80 | 0.45 | 0 | 1.00 |
| PEU2 | 5 | 5 | 5 | 4 | 5 | 5 | 4.00 | 5.00 | 4.80 | 0.45 | 0 | 1.00 |
| PEU3 | 5 | 4 | 5 | 5 | 3 | 4 | 3.00 | 5.00 | 4.40 | 0.89 | 1 | 0.80 |
| PEU4 | 5 | 4 | 5 | 5 | 4 | 5 | 4.00 | 5.00 | 4.60 | 0.55 | 0 | 1.00 |
| PEU5 | 5 | 5 | 5 | 5 | 4 | 5 | 4.00 | 5.00 | 4.80 | 0.45 | 0 | 1.00 |
| PU1 | 5 | 5 | 4 | 5 | 5 | 5 | 4.00 | 5.00 | 4.80 | 0.45 | 0 | 1.00 |
| PU2 | 5 | 5 | 4 | 5 | 5 | 5 | 4.00 | 5.00 | 4.80 | 0.45 | 0 | 1.00 |
| PU3 | 5 | 5 | 4 | 5 | 5 | 5 | 4.00 | 5.00 | 4.80 | 0.45 | 0 | 1.00 |
| PU4 | 5 | 5 | 4 | 5 | 5 | 5 | 4.00 | 5.00 | 4.80 | 0.45 | 0 | 1.00 |
| PU5 | 5 | 5 | 4 | 5 | 5 | 5 | 4.00 | 5.00 | 4.80 | 0.45 | 0 | 1.00 |
| PI1 | 5 | 5 | 5 | 4 | 3 | 4 | 3.00 | 5.00 | 4.40 | 0.89 | 1 | 0.80 |
| PI2 | 5 | 4 | 5 | 4 | 4 | 5 | 4.00 | 5.00 | 4.40 | 0.55 | 0 | 1.00 |
| PI3 | 5 | 5 | 5 | 4 | 4 | 5 | 4.00 | 5.00 | 4.60 | 0.55 | 0 | 1.00 |
| PI4 | 5 | 5 | 5 | 4 | 4 | 5 | 4.00 | 5.00 | 4.60 | 0.55 | 0 | 1.00 |
| PI5 | 5 | 4 | 5 | 4 | 5 | 5 | 4.00 | 5.00 | 4.60 | 0.55 | 0 | 1.00 |
| ADI1 | 5 | 4 | 5 | 4 | 5 | 5 | 4.00 | 5.00 | 4.60 | 0.55 | 0 | 1.00 |
| ADI2 | 5 | 5 | 5 | 5 | 4 | 5 | 4.00 | 5.00 | 4.80 | 0.45 | 0 | 1.00 |
| ADI3 | 5 | 5 | 5 | 5 | 4 | 5 | 4.00 | 5.00 | 4.80 | 0.45 | 0 | 1.00 |
| ADI4 | 5 | 5 | 5 | 5 | 4 | 5 | 4.00 | 5.00 | 4.80 | 0.45 | 0 | 1.00 |
| ADI5 | 5 | 4 | 5 | 5 | 4 | 5 | 4.00 | 5.00 | 4.60 | 0.55 | 0 | 1.00 |
| LEN1 | 5 | 4 | 5 | 5 | 3 | 4 | 3.00 | 5.00 | 4.40 | 0.89 | 1 | 0.80 |
| LEN2 | 5 | 4 | 5 | 4 | 3 | 4 | 3.00 | 5.00 | 4.20 | 0.84 | 1 | 0.80 |
| LEN3 | 5 | 4 | 4 | 4 | 4 | 5 | 4.00 | 5.00 | 4.20 | 0.45 | 0 | 1.00 |
| SRL1 | 5 | 4 | 5 | 5 | 3 | 4 | 3.00 | 5.00 | 4.40 | 0.89 | 1 | 0.80 |
| SRL2 | 5 | 4 | 5 | 4 | 4 | 5 | 4.00 | 5.00 | 4.40 | 0.55 | 0 | 1.00 |
| SRL3 | 5 | 5 | 5 | 4 | 4 | 5 | 4.00 | 5.00 | 4.60 | 0.55 | 0 | 1.00 |
| S-CVI | | | | | | | | | | | | 0.96 |

Supplementary Table 3. Reliability and Convergent Validity of Pilot Study

|  | CA | CR | AVE |
| --- | --- | --- | --- |
| ADI | 0.940 | 0.954 | 0.806 |
| LEN | 0.903 | 0.939 | 0.838 |
| PEU | 0.911 | 0.934 | 0.739 |
| PI | 0.915 | 0.936 | 0.746 |
| PU | 0.929 | 0.946 | 0.779 |
| SRL | 0.881 | 0.927 | 0.809 |

NOTE:CA = Cronbach’s alpha; CR = Composite Reliability; AVE = Average Variance Extracted

Supplementary Table 4. Fornell-Larcker Criterion of Pilot Study

|  | ADI | LEN | PEU | PI | PU | SRL |
| --- | --- | --- | --- | --- | --- | --- |
| ADI | (0.898) |  |  |  |  |  |
| LEN | 0.578 | (0.915) |  |  |  |  |
| PEU | 0.635 | 0.535 | (0.860) |  |  |  |
| PI | 0.585 | 0.635 | 0.641 | (0.864) |  |  |
| PU | 0.535 | 0.525 | 0.537 | 0.591 | (0.883) |  |
| SRL | 0.599 | 0.527 | 0.525 | 0.532 | 0.581 | (0.899) |

Supplementary Table 5. Heterotrait-Monotrait Ratio (HTMT) of Pilot Study

|  | ADI | AI | LEN | PEU | PI | PU | SRL |
| --- | --- | --- | --- | --- | --- | --- | --- |
| ADI |  |  |  |  |  |  |  |
| LEN | 0.623 | 0.036 |  |  |  |  |  |
| PEU | 0.683 | 0.041 | 0.589 |  |  |  |  |
| PI | 0.626 | 0.084 | 0.695 | 0.699 |  |  |  |
| PU | 0.568 | 0.110 | 0.569 | 0.584 | 0.638 |  |  |
| SRL | 0.653 | 0.059 | 0.591 | 0.584 | 0.590 | 0.640 |  |

Supplementary Table 6. Multi-group Test of Gender

|  | Total Effects-diff (Gender_female - Gender_male) | p-Value original 1-tailed (Gender_female vs Gender_male) | p-Value new (Gender_female vs Gender_male) |
| --- | --- | --- | --- |
| Total Effects |  |  |  |
| ADI -> LEN | 0.026 | 0.371 | 0.741 |
| ADI -> SRL | 0.148 | 0.122 | 0.244 |
| LEN -> SRL | 0.049 | 0.298 | 0.596 |
| PEU -> ADI | -0.144 | 0.907 | 0.185 |
| PEU -> LEN | -0.071 | 0.859 | 0.282 |
| PEU -> SRL | -0.018 | 0.573 | 0.854 |
| PI -> ADI | 0.064 | 0.291 | 0.582 |
| PI -> LEN | 0.041 | 0.269 | 0.538 |
| PI -> SRL | -0.173 | 0.925 | 0.149 |
| PU -> ADI | 0.107 | 0.174 | 0.348 |
| PU -> LEN | 0.065 | 0.163 | 0.326 |
| PU -> SRL | 0.043 | 0.344 | 0.688 |
| Direct Effects |  |  |  |
| ADI -> LEN | 0.026 | 0.371 | 0.741 |
| ADI -> SRL | 0.116 | 0.175 | 0.350 |
| LEN -> SRL | 0.049 | 0.298 | 0.596 |
| PEU -> ADI | -0.144 | 0.907 | 0.185 |
| PEU -> SRL | -0.036 | 0.634 | 0.732 |
| PI -> ADI | 0.064 | 0.291 | 0.582 |
| PI -> SRL | -0.220 | 0.958 | 0.083 |
| PU -> ADI | 0.107 | 0.174 | 0.348 |
| PU -> SRL | -0.016 | 0.565 | 0.870 |
| Indirect Effects |  |  |  |
| PEU -> ADI -> LEN | -0.071 | 0.859 | 0.282 |
| PI -> ADI -> LEN | 0.041 | 0.269 | 0.538 |
| PU -> ADI -> LEN | 0.065 | 0.163 | 0.326 |
| PEU -> ADI -> SRL | 0.021 | 0.341 | 0.681 |
| PI -> ADI -> SRL | 0.034 | 0.114 | 0.229 |
| PU -> ADI -> SRL | 0.041 | 0.092 | 0.185 |
| PEU -> ADI -> LEN -> SRL | -0.002 | 0.523 | 0.954 |
| PI -> ADI -> LEN -> SRL | 0.013 | 0.183 | 0.366 |
| ADI -> LEN -> SRL | 0.032 | 0.270 | 0.540 |
| PU -> ADI -> LEN -> SRL | 0.018 | 0.132 | 0.263 |

Supplementary Table 7. Multi-group Test of AI usage Frequency

|  | Total Effects-diff (Frequency_daily - Frequency_monthly) | Total Effects-diff (Frequency_daily - Frequency_weekly) | Total Effects-diff (Frequency_monthly - Frequency_weekly) | p-Value original 1-tailed (Frequency_daily vs Frequency_monthly) | p-Value original 1-tailed (Frequency_daily vs Frequency_weekly) | p-Value original 1-tailed (Frequency_monthly vs Frequency_weekly) | p-Value new (Frequency_daily vs Frequency_monthly) | p-Value new (Frequency_daily vs Frequency_weekly) | p-Value new (Frequency_monthly vs Frequency_weekly) |
| --- | --- | --- | --- | --- | --- | --- | --- | --- | --- |
| Total Effects |  |  |  |  |  |  |  |  |  |
| ADI -> LEN | 0.039 | 0.095 | 0.056 | 0.327 | 0.111 | 0.257 | 0.654 | 0.223 | 0.514 |
| ADI -> SRL | 0.036 | 0.106 | 0.070 | 0.408 | 0.222 | 0.320 | 0.817 | 0.444 | 0.640 |
| LEN -> SRL | 0.063 | -0.076 | -0.139 | 0.303 | 0.741 | 0.924 | 0.606 | 0.519 | 0.153 |
| PEU -> ADI | 0.161 | -0.027 | -0.188 | 0.162 | 0.577 | 0.910 | 0.324 | 0.847 | 0.180 |
| PEU -> LEN | 0.108 | 0.022 | -0.085 | 0.142 | 0.389 | 0.843 | 0.284 | 0.778 | 0.315 |
| PEU -> SRL | 0.118 | -0.091 | -0.209 | 0.199 | 0.787 | 0.950 | 0.399 | 0.425 | 0.101 |
| PI -> ADI | 0.120 | 0.095 | -0.026 | 0.209 | 0.198 | 0.581 | 0.418 | 0.396 | 0.838 |
| PI -> LEN | 0.080 | 0.076 | -0.004 | 0.191 | 0.141 | 0.531 | 0.381 | 0.282 | 0.938 |
| PI -> SRL | 0.145 | 0.128 | -0.017 | 0.196 | 0.178 | 0.544 | 0.392 | 0.357 | 0.912 |
| PU -> ADI | -0.237 | -0.149 | 0.088 | 0.902 | 0.884 | 0.300 | 0.195 | 0.232 | 0.599 |
| PU -> LEN | -0.132 | -0.067 | 0.065 | 0.881 | 0.816 | 0.263 | 0.238 | 0.369 | 0.526 |
| PU -> SRL | -0.308 | -0.022 | 0.286 | 0.981 | 0.579 | 0.019 | 0.038 | 0.843 | 0.038 |
| Direct Effects |  |  |  |  |  |  |  |  |  |
| ADI -> LEN | 0.039 | 0.095 | 0.056 | 0.327 | 0.111 | 0.257 | 0.654 | 0.223 | 0.514 |
| ADI -> SRL | -0.006 | 0.131 | 0.137 | 0.516 | 0.160 | 0.169 | 0.967 | 0.319 | 0.338 |
| LEN -> SRL | 0.063 | -0.076 | -0.139 | 0.303 | 0.741 | 0.924 | 0.606 | 0.519 | 0.153 |
| PEU -> ADI | 0.161 | -0.027 | -0.188 | 0.162 | 0.577 | 0.910 | 0.324 | 0.847 | 0.180 |
| PEU -> SRL | 0.058 | -0.126 | -0.184 | 0.328 | 0.852 | 0.933 | 0.657 | 0.295 | 0.135 |
| PI -> ADI | 0.120 | 0.095 | -0.026 | 0.209 | 0.198 | 0.581 | 0.418 | 0.396 | 0.838 |
| PI -> SRL | 0.100 | 0.077 | -0.023 | 0.288 | 0.303 | 0.557 | 0.576 | 0.606 | 0.885 |
| PU -> ADI | -0.237 | -0.149 | 0.088 | 0.902 | 0.884 | 0.300 | 0.195 | 0.232 | 0.599 |
| PU -> SRL | -0.244 | -0.001 | 0.243 | 0.958 | 0.499 | 0.036 | 0.084 | 0.999 | 0.071 |
| Indirect Effects |  |  |  |  |  |  |  |  |  |
| PEU -> ADI -> LEN | 0.108 | 0.022 | -0.085 | 0.142 | 0.389 | 0.843 | 0.284 | 0.778 | 0.315 |
| PI -> ADI -> LEN | 0.080 | 0.076 | -0.004 | 0.191 | 0.141 | 0.531 | 0.381 | 0.282 | 0.938 |
| PU -> ADI -> LEN | -0.132 | -0.067 | 0.065 | 0.881 | 0.816 | 0.263 | 0.238 | 0.369 | 0.526 |
| PEU -> ADI -> SRL | 0.036 | 0.047 | 0.012 | 0.275 | 0.217 | 0.440 | 0.551 | 0.433 | 0.880 |
| PI -> ADI -> SRL | 0.027 | 0.046 | 0.019 | 0.264 | 0.097 | 0.331 | 0.529 | 0.194 | 0.662 |
| PU -> ADI -> SRL | -0.056 | 0.000 | 0.056 | 0.860 | 0.529 | 0.137 | 0.280 | 0.942 | 0.274 |
| PEU -> ADI -> LEN -> SRL | 0.025 | -0.013 | -0.037 | 0.187 | 0.674 | 0.959 | 0.374 | 0.652 | 0.082 |
| PI -> ADI -> LEN -> SRL | 0.018 | 0.004 | -0.014 | 0.190 | 0.441 | 0.847 | 0.380 | 0.881 | 0.306 |
| ADI -> LEN -> SRL | 0.042 | -0.025 | -0.067 | 0.284 | 0.635 | 0.887 | 0.569 | 0.731 | 0.225 |
| PU -> ADI -> LEN -> SRL | -0.008 | -0.021 | -0.013 | 0.664 | 0.874 | 0.740 | 0.672 | 0.252 | 0.520 |

Supplementary Table 8. Common Method Bias Test with CFA Marker-variable Approach

|  | PEU | PU | PI | ADI | LEN | SRL | BSDS |
| --- | --- | --- | --- | --- | --- | --- | --- |
| PEU |  |  |  |  |  |  |  |
| PU | 0.569** |  |  |  |  |  |  |
| PI | 0.642** | 0.604** |  |  |  |  |  |
| ADI | 0.617** | 0.552** | 0.573** |  |  |  |  |
| LEN | 0.541** | 0.496** | 0.610** | 0.556** |  |  |  |
| SRL | 0.555** | 0.593** | 0.577** | 0.570** | 0.554** |  |  |
| BSDS | -0.045 | -0.04 | -0.074 | -0.006 | -0.081 | -0.066 |  |

** Correlation is significant at the 0.01 level (2-tailed).

Supplementary Table 9. Common Method Bias Test with CFA Marker-variable Approach

| Path | Original Sample (O) | Sample Mean (M) | Confidence interval | | STDEV | T | P |
| --- | --- | --- | --- | --- | --- | --- | --- |
|  |  |  | 2.5% | 97.5% |  |  |  |
| ADI -> LEN | 0.561 | 0.560 | 0.490 | 0.626 | 0.035 | 16.202 | 0.000 |
| ADI -> SRL | 0.182 | 0.182 | 0.073 | 0.297 | 0.056 | 3.242 | 0.001 |
| AI -> SRL | -0.023 | -0.024 | -0.096 | 0.046 | 0.037 | 0.628 | 0.530 |
| Frequency -> SRL | -0.037 | -0.036 | -0.110 | 0.038 | 0.038 | 0.965 | 0.335 |
| Gender -> SRL | -0.009 | -0.010 | -0.079 | 0.064 | 0.037 | 0.258 | 0.796 |
| LEN -> SRL | 0.185 | 0.184 | 0.094 | 0.268 | 0.045 | 4.153 | 0.000 |
| MV1 -> PEU | -0.045 | -0.047 | -0.148 | 0.073 | 0.057 | 0.795 | 0.427 |
| MV2 -> PU | -0.048 | -0.043 | -0.147 | 0.104 | 0.068 | 0.700 | 0.484 |
| MV3 -> PI | -0.074 | -0.075 | -0.178 | 0.042 | 0.053 | 1.400 | 0.161 |
| MV4 -> ADI | 0.019 | 0.031 | -0.052 | 0.112 | 0.042 | 0.453 | 0.651 |
| MV5 -> LEN | -0.077 | -0.079 | -0.158 | 0.013 | 0.042 | 1.819 | 0.069 |
| MV6 -> SRL | -0.027 | -0.028 | -0.094 | 0.039 | 0.034 | 0.791 | 0.429 |
| PEU -> ADI | 0.355 | 0.354 | 0.245 | 0.459 | 0.055 | 6.496 | 0.000 |
| PEU -> SRL | 0.105 | 0.104 | 0.005 | 0.201 | 0.050 | 2.098 | 0.036 |
| PI -> ADI | 0.212 | 0.213 | 0.117 | 0.307 | 0.048 | 4.403 | 0.000 |
| PI -> SRL | 0.135 | 0.136 | 0.018 | 0.261 | 0.063 | 2.156 | 0.031 |
| PU -> ADI | 0.227 | 0.228 | 0.121 | 0.338 | 0.055 | 4.139 | 0.000 |
| PU -> SRL | 0.259 | 0.260 | 0.164 | 0.355 | 0.048 | 5.402 | 0.000 |

Supplementary Table 10. Comparisons with Alternatives Model

| Model | R² | | | SRMR | NFI |
| --- | --- | --- | --- | --- | --- |
|  | ADI | LEN | SRL |  |  |
| Proposed | 0.468 | 0.315 | 0.501 | 0.070 | 0.887 |
| Alternatives Model 1 (No direct paths of ADI→SRL) | 0.464 | 0.314 | 0.478 | 0.078 | 0.894 |
| Alternatives Model 2 (LEN→ADI) | 0.313 | 0.418 | 0.493 | 0.110 | 0.892 |
